# Exploring the exercise for enhancing postural control, gait, and muscle strength in older adults with diabetic peripheral neuropathy: a systematic review and meta-analysis

**DOI:** 10.3389/fragi.2025.1507232

**Published:** 2025-04-29

**Authors:** Xiangsheng Pang, Dongmei Wang, Fei Zhang, Bin Guo, Wenming Liu

**Affiliations:** ^1^ Department of Sports Science, Zhejiang University, Hangzhou, Zhejiang, China; ^2^ Sport Science School, Beijing Sport University, Beijing, China; ^3^ College of Physical Education, Hangzhou Normal University, Hangzhou, Zhejiang, China; ^4^ School of Physical Education, Da Lian University, Dalian, Liaoning, China

**Keywords:** diabetic peripheral neuropathy, gait characteristics, muscle strength, postural control, fall prevention

## Abstract

**Objective:**

The purpose of this study was to conduct a systematic review and meta-analysis to evaluate the effects of exercise on postural control, gait, and muscle strength in older adults with diabetic peripheral neuropathy (DPN).

**Research Design:**

Systematic review and meta-analysis.

**Methods:**

An extensive literature search was performed in PubMed, EBSCO, Web of Science and Cochrane Library from database inception to 30 September 2023. The inclusion criteria were exercise intervention on postural control, gait characteristics, and muscle strength in older adults with DPN. Two reviewers independently extracted data and assessed the quality of studies by Cochrane Risk of Bias.

**Results:**

The literature search elicited a total of 523 references, 23 articles were included in this systematic review and meta-analyses. Exercise could effectively decrease the Centre of Pressure (COP) path (SMD = −0.38, 95%CI = −0.77 ∼ 0.01), increase gait speed (MD = 0.08, 95%CI = 0.05 ∼ 0.11), but did not change stride length (MD = 0.04, 95%CI = −0.01 ∼ 0.09), and enhance muscle strength (SMD = 0.76, 95%CI = 0.19 ∼ 1.33).

**Conclusion:**

Exercise improves postural control, gait speed, and muscle strength in older adults with DPN, reducing fall risk and enhancing lower limb strength, though evidence on stride length improvement is limited.

**Systematic Review Registration:**

identifier CRD42023436799.

## 1 Introduction

Diabetic Peripheral Neuropathy (DPN), a prevalent chronic complication of diabetes, significantly impacts the quality of life in approximately 50% of diabetic patients (Sloan et al., 2021). The pathogenesis of DPN is multifaceted, primarily attributed to prolonged hyperglycemia and metabolic dysregulation, leading to sensory and motor nerve damage (Sloan et al., 2021). Notably, individuals with DPN exhibit a 2.3-fold higher risk of falls compared to diabetic patients without DPN (Reeves et al., 2021; Kruse et al., 2010), and a staggering 15-fold increase relative to their healthy counterparts (Khan et al., 2021a; Mustapa et al., 2016). Clinical manifestations of DPN include distal sensory abnormalities, neuropathic pain, muscle weakness, and motor dysfunction, collectively contributing to gait disturbances and impaired postural control (Sloan et al., 2021). These impairments substantially elevate fall risk, with potentially severe consequences such as fractures and intracranial hemorrhages, which not only impose significant economic burdens but are also associated with increased mortality rates (Gupta et al., 2023).

A complex interaction of factors affects the increased risk of falls among older adults with DPN. This is attributed to the glycation of skeletal muscle proteins as well as axonal degeneration and segmental demyelination of the peripheral motor nerve (Reeves et al., 2021), leading to lower extremity motor impairments and a loss of sensory feedback in the feet (Reeves et al., 2021). DPN causes pain, diminished muscle quality, diminished peripheral sensation, unstable gait, impaired balance, and motor dysfunction, ultimately resulting in increased fall risk (Khan et al., 2021b). In particular, postural instability and gait imbalance in DPN mainly contribute to high fall incidence (Wang et al., 2022). People with DPN usually exhibit a conservative gait strategy with worse gait speed and step length (Reeves et al., 2021). Reeves et al. reported the strongest correlations with individuals’ self-perceived unsteadiness were with gait velocity, stride length and severity of DPN (Reeves et al., 2017). Otherwise, it is possibly due to the part absence of peripheral sensation and the delaying of neuromuscular control this could result in balance impairment and a high risk of falls in the medial-lateral (ML) dynamic sway (Brown et al., 2015), as a key indicator to distinguish among people with and without DPN (Reeves et al., 2017).

Exercise intervention, as a first-line non-pharmacological treatment strategy for diabetic peripheral neuropathy (DPN), holds significant potential in enhancing patients’ postural control, gait function, and muscle strength (Johnson and Takemoto, 2019). These interventions include balance training (Song et al., 2011; Lee et al., 2013; Grewal et al., 2015), resistance training (Melai et al., 2014; Sartor et al., 2014), aerobic training (Abdelaal and El-Shamy, 2022; Zhao et al., 2021), multicomponent exercise (Perrin et al., 2021; Waheed et al., 2021), and Tai Chi, which can effectively control blood glucose levels and thereby reverse motor dysfunction caused by neuropathy (Sloan et al., 2021). Improving balance ability in older adults is often a primary goal of fall prevention interventions (Blodgett et al., 2022). Research by Ahmad and colleagues further confirmed that an eight-week sensorimotor training intervention significantly improved patients’ balance and proprioception (Ahmad et al., 2019). Aerobic training not only enhances neural structure and function but also alleviates neuropathic signs and symptoms (Perrin et al., 2021; Dixit et al., 2016). Additionally, physical exercise can reduce pain and/or numbness caused by neuropathy, ultimately improving instability and mobility (Li and Hondzinski, 2012). A review noted that exercise combined with dietary interventions can induce systemic and cellular changes, thereby ameliorating complications associated with DPN (Enders et al., 2023). Although exercise can improve balance, reduce fear of falling, and enhance the quality of life (Lima et al., 2021), there is currently no consensus on the extent to which exercise improves postural control, gait characteristics, and muscle strength in older adults with DPN. Furthermore, it remains unclear whether these improvements contribute to reducing the risk of falls in this population.

This study conducted a systematic review and meta-analysis of randomized controlled trials to assess the outcomes of exercise interventions on fall risk factors including postural control, gait and muscle strength in older adults with DPN.

## 2 Methods

### 2.1 Protocol and registration

The review study followed the Preferred Reporting Items for Systematic Review and Meta-Analyses (PRISMA) 2020 statement (Page et al., 2021). A protocol was registered in the International Prospective Register of Systematic Reviews (PROSPERO) ID: CRD42023436799.

### 2.2 Eligibility criteria

The PICOS (populations, interventions, comparator interventions, outcomes and study design) framework guided the eligibility criteria selection (Cochrane Collaboration, 2024).

#### 2.2.1 Populations

The diagnosis of DSPN follows an exclusion-based approach, with diagnostic criteria encompassing the following key points: (1) a confirmed history of diabetes; (2) onset of neuropathy at or after the diagnosis of diabetes; (3) presence of clinical symptoms of neuropathy (e.g., pain, numbness, paresthesia) accompanied by at least one abnormal finding in five neurological examinations (ankle reflex, vibration sensation, pressure sensation, temperature sensation, and pinprick sensation); in the absence of clinical symptoms, at least two abnormal findings are required; (4) exclusion of other potential causes of neuropathy, including neurotoxic medications (e.g., chemotherapeutic agents), vitamin B12 deficiency, cervical or lumbar spine disorders (e.g., compression, stenosis, degenerative changes), cerebral infarction, chronic inflammatory demyelinating polyneuropathy, hereditary neuropathies, vasculitis, infections (e.g., acquired immunodeficiency syndrome), and metabolic neurotoxicity secondary to renal insufficiency.

#### 2.2.2 Interventions

The included interventions in this study primarily encompass aerobic exercise, anaerobic training, resistance exercise, whole-body vibration training, balance exercise, sensorimotor training, foot-ankle functional exercise, Tai Chi, yoga, dance training, as well as combinations of two or more of the aforementioned exercise modalities. Exercise combined with other non-exercise interventions, such as co-administration of medication, acupuncture, electrical stimulation, heat application, or physical therapy, or lifestyle modifications, have been excluded. Moreover, studies with fully supervised exercise programs or those involving short-term exercise interventions (<1 week) have also been excluded.

#### 2.2.3 Comparator interventions

The control group interventions may include no exercise, routine foot care, health education, or regular physical activity interventions.

#### 2.2.4 Outcomes

The outcome of the study was one or more of the following: (1) postural control: COP sway path; (2) gait parameters: speed, stride length, step cycle time, cadence, etc.; (3) muscle strength.

#### 2.2.5 Studies design

This study included RCTs on exercise interventions for older adults with DPN in all settings (community, hospitals and institutions). It is acceptable that this literature only considered exercise as the exposure or intervention factor.

### 2.3 Information sources and search strategy

Three-step search for relevant randomized controlled trials (RCTs) as recommended by the Cochrane Handbook for Systematic Reviews of Interventions was conducted (Cochrane Collaboration, 2024). Four electronic databases (PubMed, Web of Science, EBSCO, Cochrane Library) were searched for articles published up to 9 September 2023. Then, searching was also done in published trial articles. Medical Subject Headings (MeSH) terms and keywords were chosen based on study design (“Randomized Controlled Trials”), exposure (“Exercise” OR “Training” OR “Physical activity”), outcomes (“Postural Control” OR “Gait Performance” OR “Biomechanics”) and participants (“Diabetic Peripheral Neuropathy”). The full search strategy for PubMed can be found in the online Supplementary Appendix B. Reference lists of included studies were also searched for relevant articles.

### 2.4 Study selection

All returned titles were screened by the first author (DW) to exclude duplicate or non-relevant studies. The abstract of each remaining study was then independently reviewed by DW and XP during the literature search. Then the full texts of the remaining studies were independently reviewed by the two authors against the inclusion and exclusion criteria. Disagreements were discussed and consensus was reached among the authors in all cases. All studies in the systematic review were eligible for inclusion in the meta-analyses.

### 2.5 Data collection process and data items

We collected data on authors, year of publication, number of participants allocated to the intervention and control groups, participant-based information (age, gender, BMI, duration of diabetes, HbA1c), type and duration of exercise intervention for the experimental and control groups (time, frequency), outcome measures, and study duration. This study gathered the outcomes including RCTs in exercise intervention, encompassing statistical metrics of continuous variables such as sample size, mean, standard deviation, and others for each study.

### 2.6 Study risk of bias assessment

Two authors (DW and XP) independently assessed the risk of bias at the study level of included RCTs following the Cochrane Risk of Bias Tool (RoB 2) (Sterne et al., 2019). The seven items considered for the risk of bias included: the randomization process, bias arising from period and carryover effects, deviations from intended interventions, missing outcome data, measurement of the outcome, selection of the reported result and overall bias. Open and apply the macro using the Excel tool (website of Cochrane Methods Bias: https://www.riskofbias.info/) to assess the risk of bias for each article (Liu et al., 2021).

### 2.7 Synthesis methods

This study employed RevMan 5.3 (The Cochrane Collaboration, Copenhagen, Denmark) software to perform meta-analysis, subgroup analysis, and generate forest plots. The extracted data were all continuous variables, expressed as mean difference (MD) and their 95% confidence intervals (CI). If the units were inconsistent, standardized mean difference (SMD) was used. Heterogeneity of outcome measures was assessed using the I^2^ statistic and p-values. If heterogeneity was low (I^2^ ≤ 50%, p ≤ 0.01), a fixed-effects model was applied; if heterogeneity was high (I^2^ > 50%, p > 0.01), a random-effects model was used (Higgins et al., 2003). Subgroup analyses focused on three key factors: postural control, gait, and muscle strength. Each factor was further subdivided based on different variables to minimize heterogeneity in the study. If significant heterogeneity persisted, sensitivity analysis was conducted to identify its sources. If the sources of heterogeneity could not be determined, descriptive analysis was performed. The significance level *ɑ* for the pooled effect size was set at 0.05.

## 3 Results

### 3.1 Study selection

**FIGURE 1:**
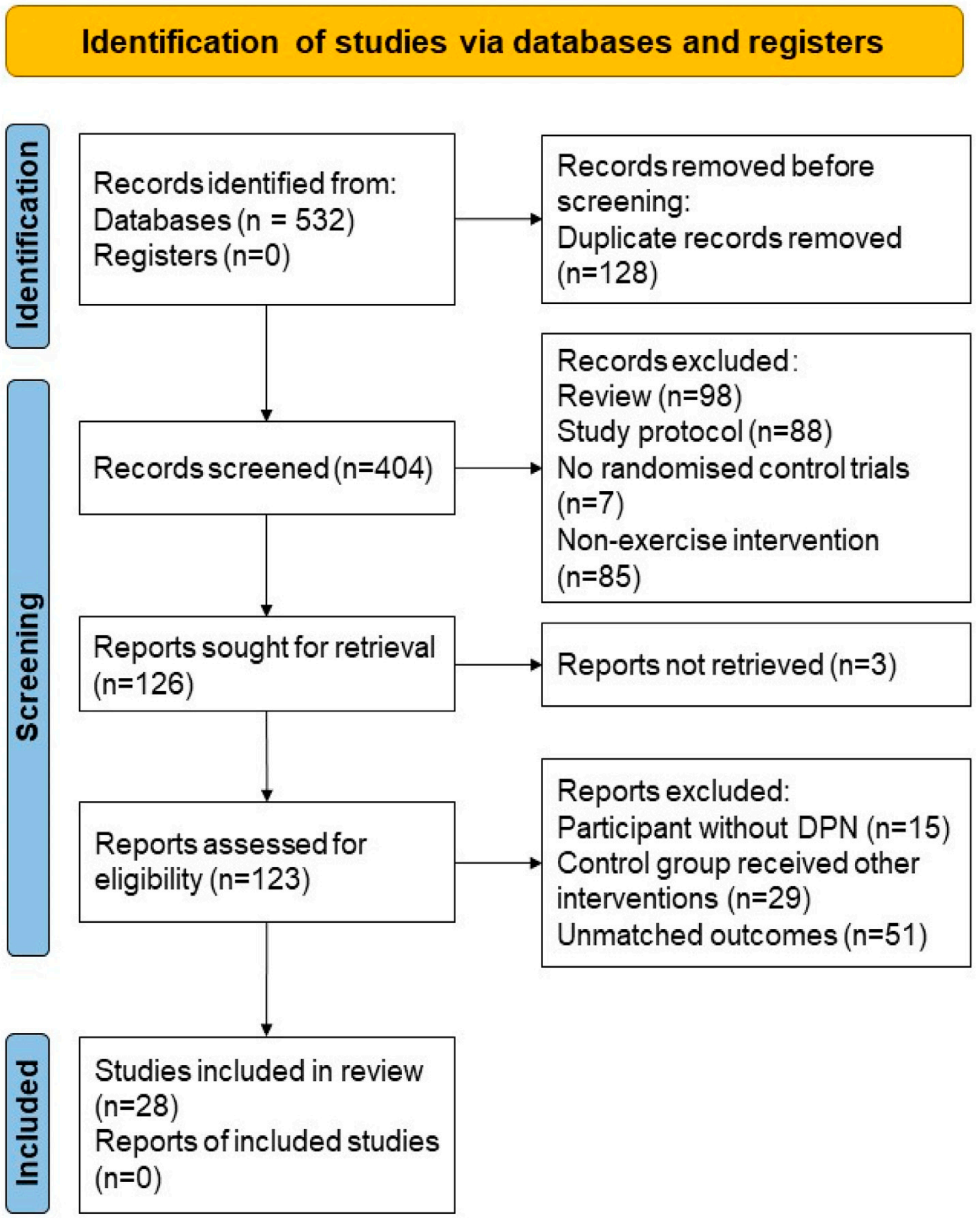
Study selection based on PRISMA 2020 flow diagram.

After the initial search, a total of 532 articles were included. After removing 128 duplicate articles, 280 articles were excluded based on title and abstract screening. Further full-text retrieval, reading, and quality assessment led to the exclusion of 98 articles that did not meet the inclusion criteria. Ultimately, 23 articles were included in the study. The process of article inclusion and exclusion is illustrated in Figure 1. The characteristics of the study participants, intervention, control, and outcome measures were shown in the review (Table 1).

**TABLE 1:** Characteristics of the included studies, including type and duration of exercise, control intervention, and outcome measure.

Reference (year)	Exercise	Control	Type and duration of exercise	Control intervention	Outcome measures
Allet et al. (2010)	N = 35	N = 36	Balance exercise (12 weeks, 2 days/week)	None	Gait velocity, Cadence, Gait cycle time, Stride length, Stance time percentage
Kruse et al. (2010)	N = 41	N = 38	Strength and balance exercise (12 weeks, 8 sessions)	Medical care	Ankle dorsiflexion strength
Song et al. (2011)	N = 19	N = 19	Balance exercise (8 weeks, 2 days/week)	Health education (8 weeks, 1 day/week)	COP sway path, 10-m walk time
Lee et al. (2013)	N = 37	N = 18	1: Whole-body vibration training (6 weeks, 3 days/week) and balance exercise (6 weeks, 2 days/week); 2: Balance exercise (6 weeks, 2 days/week)	None	COP sway velocity, FTSTS
Mueller et al. (2013)	N = 15	N = 14	Strength and aerobic exercise (12 weeks, 3 days/week)	Aerobic Exercise (12 weeks, 3 days/week)	Six-minute Walking Test
Melai et al. (2014)	N = 48	N = 46	Strength training (24 weeks, 2 days/week)	None	Gait velocity, Stride length, Stance phase duration, Stride time
Sartor et al. (2014)	N = 26	N = 29	Strength training (12 weeks, 2 days/week)	None	COP sway velocity, Muscle function
Yoosefinejad et al. (2015)	N = 10	N = 10	Whole-body vibration training (6 weeks, 2 days/week)	None	COP sway velocity, Quadriceps strength, Tibialis Anterior strength, General strength
Dixit et al. (2016)	N = 36	N = 46	Aerobic exercise (8 weeks, 3–6 days/week)	Health education	COP sway velocity, COP sway displacement
Ahmad et al. (2019)	N = 20	N = 17	Sensorimotor training (8 weeks, 3 days/week)	Health education	COP range, COP sway path, FRT
Venkataraman et al. (2019)	N = 70	N = 73	Strength and balance exercise (8 weeks, 1 day/week)	Routine clinical care	COP sway velocity, Ankle muscle strength, FTSTS
Abdelbasset et al. (2020)	N = 14	N = 14	Proprioceptive training (8 weeks, 3 days/week)	None	Six-minute Walking Test
Monteiro et al. (2020)	N = 15	N = 15	Foot-ankle exercise (12 weeks, 2 days/week)	Usual Care	Gait velocity, Hallux maximum force, Toe maximum force
Ahmad et al. (2021)	N = 32	N = 26	Sensorimotor training (8 weeks, 3 days/week)	Health education	Gait velocity, Cadence, Stride length, Single stance support, Double limb support
Perrin et al. (2021)	N = 12	N = 12	Balance and proprioceptive exercise (8 weeks, 3 days/week)	Usual Care	FTSTS
Zhao et al. (2021)	N = 20	N = 20	Tap dance training (16 weeks, 3 days/week)	Educational workshops (1 session/month)	COP trajectory, COP elliptical area, FTSTS
Abdelaal and El-Shamy (2022)	N = 23	N = 22	Antigravity treadmill training and traditional physical therapy (12 weeks, 3 days/week)	Traditional physical therapy (12 weeks, 3 days/week)	Step length, Step time, Double support time, Gait velocity, Cadence
Khan et al. (2022)	N = 15	N = 15	Strength exercise (12 weeks, 2–3 days/week)	None	Gait speed
Monteiro et al. (2022)	N = 39	N = 39	Foot-ankle exercise (12 weeks, 4 days/week)	Usual Care	Gait velocity, Hallux strength, Toes strength
Rodríguez-reyes et al. (2022)	N = 20	N = 21	Whole-body vibration training (12 weeks, 3 days/week)	Usual care	Gait speed
Waheed et al. (2021)	N = 19	N = 19	Whole-body vibration and balance exercise (3 weeks, 5 days/week) and dietary advice (1 day/week)	Balance exercise (3 weeks, 5 days/week) and dietary advice (1 day/week)	Quadriceps strength, Tibialis anterior strength
Hizomi arani et al. (2023); Silva et al. (2021)	N = 22	N = 22	Foot-ankle exercise (8 weeks, 3 days/week)	None	Gait velocity
Silva et al. (2023)	N = 25	N = 25	Foot-related exercise (8 weeks, 3 days/week)	Usual care	Hallux strength, Toes strength

### 3.2 Risk of bias in the included studies

The 10 studies included in this research all exhibited a low risk in the randomization process (Kruse et al., 2010; Song et al., 2011; Lee et al., 2013; Sartor et al., 2014; Waheed et al., 2021; Dixit et al., 2016; Khan et al., 2022; Monteiro et al., 2022; Hizomi arani et al., 2023; Silva et al., 2023), with no instances of missing data. Among these, one study was an open-label randomized controlled trial (Rodrigues et al., 2022), resulting in a high risk in intervention allocation; two studies did not implement allocation concealment for participants or assessors (Melai et al., 2014; Venkataraman et al., 2019), leading to some risk in intervention allocation; and two studies failed to conceal allocation from treatment providers, leading to a high risk of bias in intervention compliance (Rodrigues et al., 2022; Yoosefinejad et al., 2015). The ten studies have not clearly described the concealment of the allocation of treatment and testing personnel, introducing some risk to intervention adherence (Melai et al., 2014; Abdelaal and El-Shamy, 2022; Zhao et al., 2021; Perrin et al., 2021; Waheed et al., 2021; Ahmad et al., 2019; Venkataraman et al., 2019; Allet et al., 2010; Mueller et al., 2013; Abdelbasset et al., 2020). The risk of bias assessment results for the included studies are presented in Table 2.

**TABLE 2:** Risk of bias summary.

Reference (year)	Randomization process	Bias arising from period and carryover effects	Deviations from intended interventions	Missing outcome data	Measurement of the outcome	Selection of the reported result	Overall bias
Allet et al. (2010)	L	L	S	L	L	L	S
Kruse et al. (2010)	L	L	L	L	L	L	L
Song et al. (2011)	L	L	L	L	L	L	L
Lee et al. (2013)	L	L	L	L	L	L	L
Mueller et al. (2013)	L	L	S	L	L	L	S
Melai et al. (2014)	L	S	S	L	L	L	S
Sartor et al. (2014)	L	L	L	L	L	L	L
Yoosefinejad et al. (2015)	L	L	H	L	L	L	H
Dixit et al. (2016)	L	L	L	L	L	L	L
Ahmad et al. (2019)	L	L	S	L	L	L	S
Venkataraman et al. (2019)	L	S	S	L	L	L	S
Abdelbasset et al. (2020)	L	L	S	L	L	L	S
Monteiro et al. (2020)	L	L	L	L	L	L	S
Ahmad et al. (2021)	L	L	S	L	L	L	S
Perrin et al. (2021)	L	L	S	L	L	L	S
Zhao et al. (2021)	L	L	S	L	L	L	S
Abdelaal and El-Shamy (2022)	L	L	S	L	L	L	S
Khan et al. (2022)	L	L	L	L	L	L	L
Monteiro et al. (2022)	L	L	L	L	L	L	L
Rodríguez-reyes et al. (2022)	S	L	H	L	L	L	H
Waheed et al. (2021)	L	L	L	L	L	L	L
Hizomi arani et al. (2023); Silva et al. (2021)	L	L	L	L	L	L	L
Silva et al. (2023)	L	L	L	L	L	L	L

L: low risk of bias; S: some concerns bias; H: high risk of bias.

### 3.3 Outcome measures

#### 3.3.1 COP sway path

**FIGURE 2:**
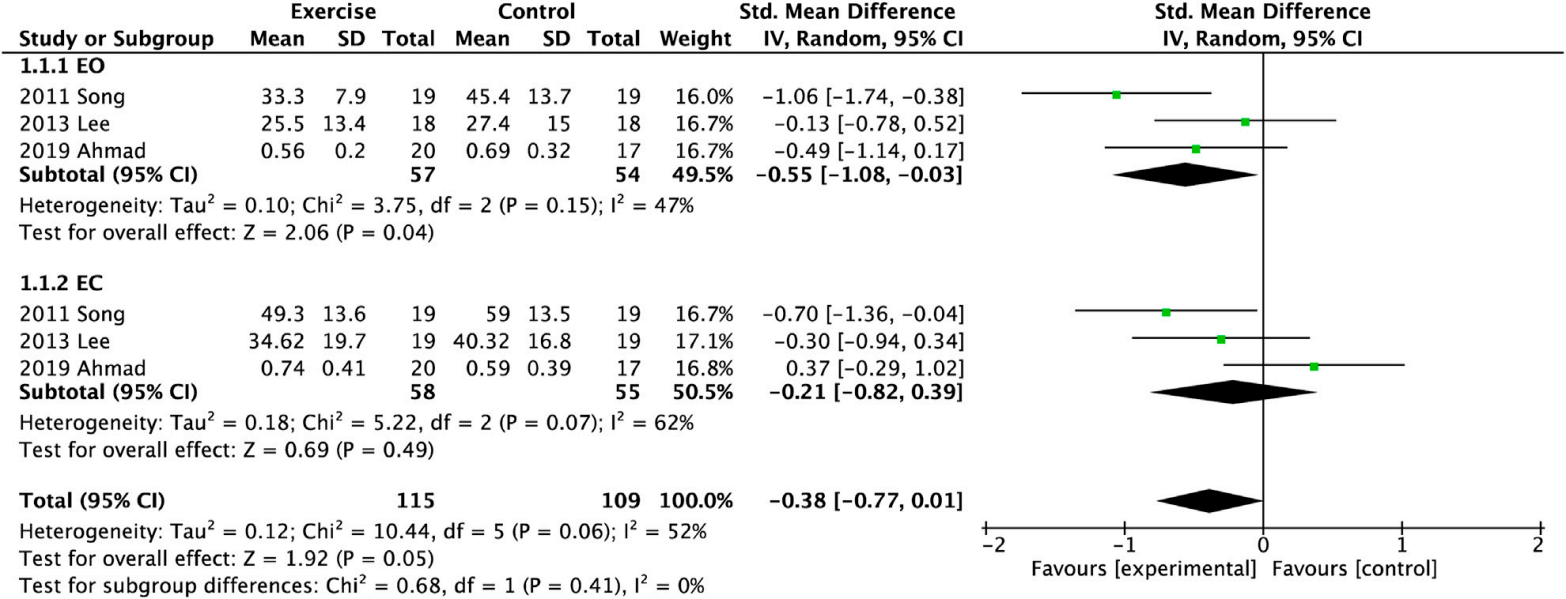
Forest plot of meta-analyses showing the effect of centre of pressure (COP) sway path with open eye (EO) and closed eye (EC).

There were three studies regarding the COP sway path after exercise in the AP and ML directions (Figure 2). Overall, it was shown that exercise could effectively decrease the sway path of postural control (SMD = −0.38, 95%CI = −0.77 ∼ 0.01). In particular, the pooled results showed significant differences with EO (SMD = −0.55, 95%CI = −0.108 ∼ −0.03), but no difference with EC (SMD = −0.21, 95%CI = −0.82 ∼ 0.39). The total heterogeneity was low to moderate (I^2^ = 28%, $\chi^{2}$ = 12.55).

#### 3.3.2 Gait characteristics

**FIGURE 3:**
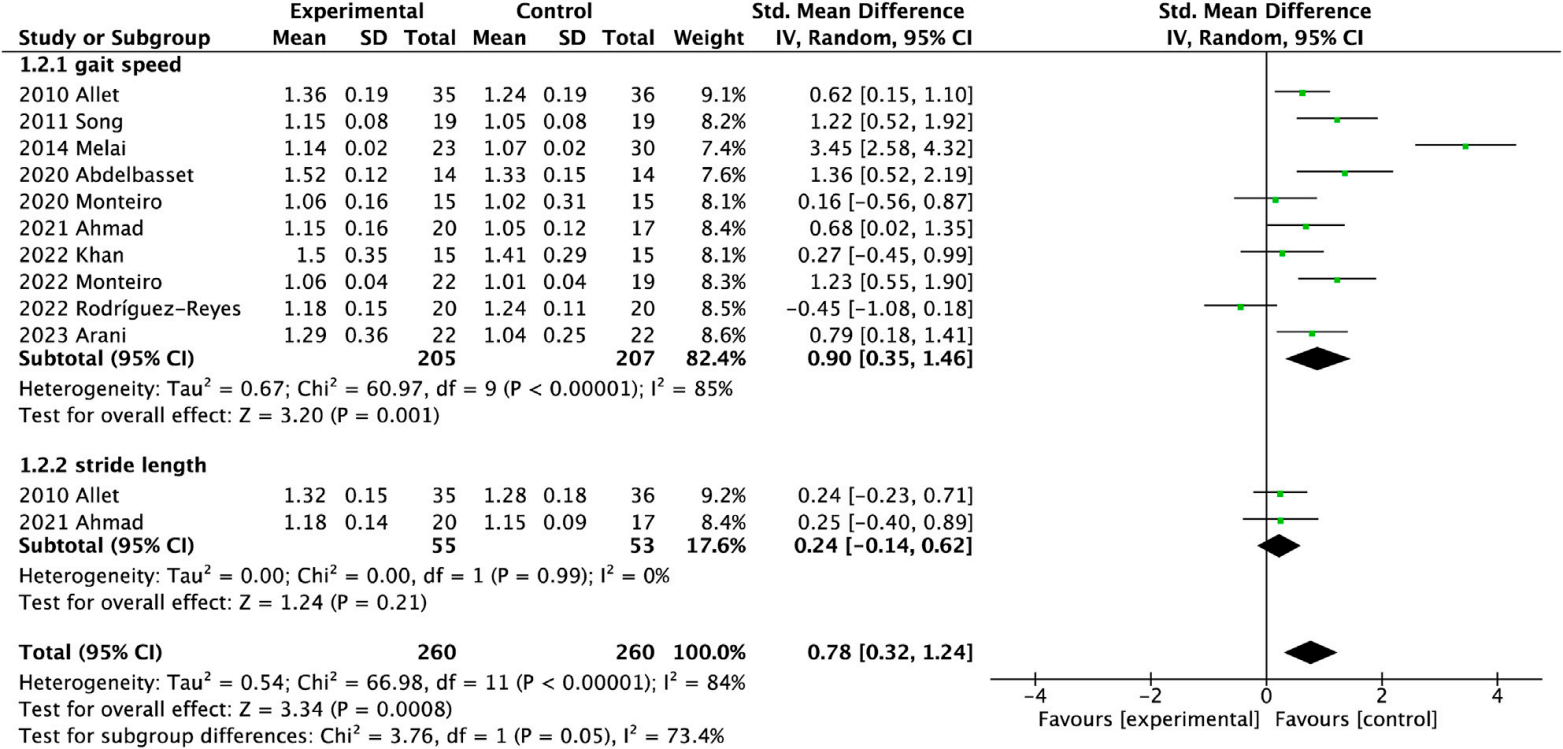
Forest plot of meta-analyses showing the effect of gait speed and stride length.

There were ten studies on gait speed involving 412 participants and three studies on stride length involving 145 participants in Figure 3-1.2.1. Gait performance was measured with the gait velocity and stride length. Gait velocity had significant differences between the interventional and control groups (MD = 0.08, 95%CI = 0.05 ∼ 0.11), with moderate heterogeneity (I^2^ = 63%, $\chi^{2}$ = 24.55). There were two studies on stride length involving 145 participants in Figure 3-1.2.2. Stride length was not increased after exercise (MD = 0.04, 95%CI = −0.01 ∼ 0.09), with related low heterogeneity on the whole (I^2^ = 0%, $\chi^{2}$ = 0.10).

#### 3.3.3 Muscle strength

**FIGURE 4:**
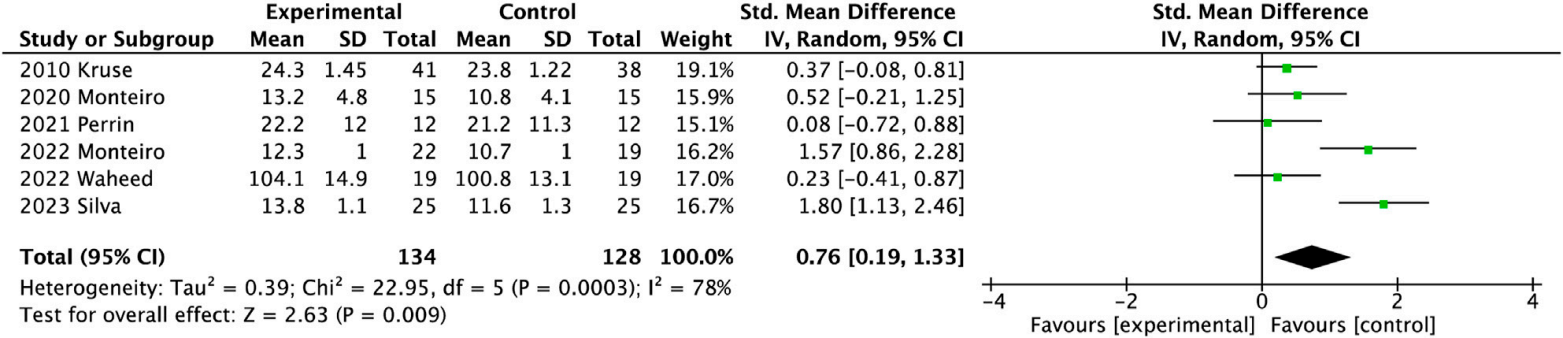
Forest plot of meta-analyses showing the effect of muscle strength.

As shown in Figure 4, six studies involving 262 participants presented the muscle strength of lower limbs. The muscle strength of the lower extremity was significantly reduced after exercise (SMD = 0.76, 95%CI = 0.19 ∼ 1.33).

## 4 Discussion

Exercise, as a clinical rehabilitation tool, can partially restore sensorimotor impairments accompanied by neuropathic symptoms and promote physical function. This review aims to evaluate the effectiveness of exercise interventions on postural control, gait characteristics, and muscle strength in older adults with DPN, and to provide evidence-based exercise recommendations.

### 4.1 Effect of exercise on postural control

Impaired Balance is a strong risk factor for falls with a medium to large effect size (Chantanachai et al., 2021). In previous studies, these methods of balance testing had a high heterogeneity within or across settings (Perell et al., 2001), such as COP, TUG, BBS, OLS, and so on (Blodgett et al., 2022; Perell et al., 2001). Objective evaluation of postural control is usually based on the analysis COP using a force platform (Blodgett et al., 2022; Quijoux et al., 2020), as the gold standard for evaluation of balance. The velocity and sway area of COP were the best features for discriminating between fallers and non-fallers (Perell et al., 2001), and had a high correlation with the severity of neuropathy (Brown et al., 2015; Perell et al., 2001). In this review, three studies (Melai et al., 2014; Enders et al., 2023) included meta-analysis to estimate postural control between experimental and control groups and observed that could significantly decrease AP sway amplitude with EO and EC after balance training. Although studies presented stability in the ML direction as a key factor to differentiate high fall risk among older population (Brown et al., 2015), Figure 2 showed only COP sway path with EC had improved compared with EO.

Otherwise, five studies with other exercises were not included in the meta-analysis (Sartor et al., 2014; Zhao et al., 2021; Dixit et al., 2016; Venkataraman et al., 2019; Yoosefinejad et al., 2015). Dixit and coworkers only found that eight-week aerobic exercise could reduce COP sway velocity along the x-axis and increase ML displacement on foam with EC (Dixit et al., 2016). Zhao et al. also validated the effect of aerobic exercise significantly affected COP trajectory and elliptical area (Zhao et al., 2021). A study described a relatedly lower COP velocity measured by plantar pressure system after 12-week strength training (Sartor et al., 2012), but the number and quality of each study were relatively less. However, a meta-analysis reported that balance exercise intervention changed the COP parameters in either eyes or closed condition among older adults (age > 60), while resistance and multi-component exercise did not (Low et al., 2017). What are the possible explanations for the relatively fewer changes observed in COP sway indicators of ML direction? A study showed that ML sway path length/velocity did not change in older adults after balance exercises (Low et al., 2017), similar to this review. Moreover, another review showed that ML data were found to be more discriminatory than AP features (Quijoux et al., 2020). The possible reasons were the small sample size and inconsistencies in the data collection protocols, leading to high heterogeneity (69%) of the ML sway path.

In conclusion, exercise interventions revealed a moderate to high effect on balance performance parameters compared to the control group, and balance training significantly improved postural control to a greater extent compared to other exercises. Balance exercise, in this review, included regular stability training, sensorimotor training, proprioception training, and combined training above. A review presented that sensorimotor training also plays a crucial role in targeting balance control and existing sensory and motor signs and symptoms of DPN (Streckmann et al., 2022).

### 4.2 Effect of exercise on gait performance

It is well known that gait performance, a natural daily activity heavily reliant on the synergy between the nervous and musculoskeletal systems, can be easily affected by the pathological process in older adults with DPN (Wang et al., 2022). Adults with DPN usually adopt a conservation gait with lower gait velocity, shorter stride length, longer stride time and stance time, compared with non-neuropathy diabetes (Wang et al., 2022). A study showed that exercise could significantly improve gait velocity compared to a control group, especially balance exercise (Allet et al., 2010). Some described that velocity and stride length effectively increased after 8-week sensorimotor exercise in older adults with DPN regardless of age (Reeves et al., 2021), similar to this review. In general, studies reported that lower walking speeds accompanied by increased falling risk in older adults, significantly discriminate between fallers and non-fallers (Reeves et al., 2017). Based on meta-analysis, balance exercise could more greatly increase gait velocity under both self-gait speed and fast-gait speed conditions than resistance training. Otherwise, this review reported that stride length only had an increase trend after exercise interventions. There was a potential factor that the sample size was small only 75 in the interventional group and 70 in the control group. Another reason was that cadence contributed 80% whereas stride length only contributed 20% to this change of gait velocity, resulting in difficulty regulating stride length (Allet et al., 2010).

### 4.3 Effect of exercise on muscle strength

Diabetes is responsible for the deterioration of muscle strength and becomes more severe as DPN progresses, leading to altered gait biomechanics, impaired stability and increased fall risks (Orlando et al., 2022). Some suggested that reduced strength of the knee and ankle may cause a disturbance in perturbation response in balance, potentially increasing the risk of falling. Although these results had a low level of quality and evidence, in this review, exercise could increase muscle strength and joint mobility in people with DPN, especially after foot-ankle functional training. Regarding the foot-ankle exercise, the exercise protocol is designed to consist of the same set: warm-up exercise, strengthening of the intrinsic foot muscles, strengthening of the extrinsic foot muscles, and functional exercise (e.g., balance and gait training), to manage the musculoskeletal complications related to diabetes (Silva et al., 2023; Monteiro et al., 2020). The observed phenomenon of reducing foot-ankle joint mobility and intrinsic muscle strength may be attributed to symptoms of distal peripheral neuropathy (Mustapa et al., 2016; Wang et al., 2022). Strength combined with functional training provided a better effect on increasing muscle strength of the lower extremity, compared to resistance training alone. Although resistance exercise was an effective intervention to combat muscle loss and delay some neurological symptoms (Melai et al., 2014; Venkataraman et al., 2019), people with neuropathy should be wary of weight-bearing exercise because of plantar loading links to potential foot ulcer development (Li and Hondzinski, 2012). Combinational exercises could more greatly improve metabolic control of the organism than aerobic and resistance training, similar to the previous study (Orlando et al., 2022). Improving foot functionality has a positive impact on people’s overall physical activity levels and quality of life (Streckmann et al., 2022). Foot-ankle functional training significantly increased ankle joint ranges of motion including dorsiflexion and plantarflexion in older adults with DPN. That is the foot-ankle exercise program focused mainly on the foot joints of muscle strength and mobility in gait or dynamic activities.

### 4.4 Limitations of the study

This review has some limitations. The primary limitation is the focus on short-term outcomes, without accounting for the potential long-term benefits or risks associated with the interventions. Moreover, this study did not conduct subgroup analyses based on exercise types or exercise prescriptions (such as intensity, duration, frequency, etc.), and failed to provide detailed guidance for the implementation of specific exercise programs in the daily rehabilitation of DPN patients. Furthermore, although 23 studies were included in this review, the exercise modalities assessed were not evenly distributed. Most studies focused on balance training, followed by multi-component and foot-ankle functional exercises. In contrast, strength training and whole-body vibration training were represented by fewer studies. This uneven distribution may contribute to increased heterogeneity across the meta-analysis and limit the precision of the confidence intervals. Therefore, clinicians should interpret the results with caution when considering them for decision-making.

## 5 Conclusion

Exercise is a fundamental intervention for older adults with DPN and significantly improves physical activity, measured by postural control, gait characteristics and muscle strength. Exercise could enhance postural control under the open eye and closed eye to prevent or reduce the risk of falls. Gait is a component of ability and skill for daily life. Exercise could effectively decrease the gait speed in walking, but did not improve the stride length with low-quality evidence. This study found that the muscle strength in the lower limbs was significantly enhanced after exercise among older adults with DPN.

## Data Availability

The raw data supporting the conclusions of this article will be made available by the authors, without undue reservation.
